# Non-coding RNA and hepatitis B virus-related hepatocellular carcinoma: A bibliometric analysis and systematic review

**DOI:** 10.3389/fmed.2022.995943

**Published:** 2022-09-20

**Authors:** Li-rong Yan, Ao-ran Liu, Li-yue Jiang, Ben-gang Wang

**Affiliations:** ^1^Tumor Etiology and Screening Department of Cancer Institute and General Surgery, The First Hospital of China Medical University, Key Laboratory of Cancer Etiology and Prevention, China Medical University, Liaoning Provincial Education Department, Shenyang, China; ^2^Tangdu Hospital of the Fourth Military Medical University, Xi’an, China; ^3^Department of Hepatobiliary Surgery, Institute of General Surgery, The First Hospital of China Medical University, Shenyang, China

**Keywords:** non-coding RNA, HBV-related hepatocellular carcinoma, bibliometric analysis, long noncoding RNA (LncRNA), microRNA (miRNA)

## Abstract

**Objectives:**

A bibliometric analysis for non-coding RNA and hepatitis B virus (HBV)-related hepatocellular carcinoma (HCC) was performed to describe international research status and visualize the research scope and emerging trends over the last two decades on this topic.

**Materials and methods:**

Research data of non-coding RNA and HBV-related HCC were retrieved and extracted from the Web of Science Core Collection (WoSCC) database from 1 January 2003 to 13 June 2022 and then analyzed by means of bibliometric methods. A total of 1,036 articles published in this field were assessed for specific characteristics, including the year of publication, journal, author, institution, country/region, references, and keywords. VOSviewer was employed to perform co-authorship, co-occurrence, and co-citation analyses accompanied by constructing a visual network.

**Results:**

Overall, 1,036 reports on non-coding RNA and HBV-related HCC from 2003 to 2022 were retrieved from WoSCC. The publication has gradually increased during the last two decades with 324 journals involved. Most research records (748 publications and 23,184 citations) were concentrated in China. A co-occurrence cluster analysis for the top 100 keywords was performed and four clusters were generated: (1) non-coding RNA as a molecular marker for the diagnosis and prognosis of HBV-related HCC; (2) dysregulation of non-coding RNA by hepatitis B virus X protein (HBx); (3) non-coding RNA affecting the biological behaviors of HBV-related HCC; and (4) epidemiological study for the effects of non-coding RNA on the risk of HBV-related HCC.

**Conclusion:**

The publications and citations involved in non-coding RNA and HBV-related HCC have increased over the last two decades associated with many countries, institutions, and authors. Our study revealed current development trends, global cooperation models, basic knowledge, research hotspots, and emerging frontiers in this field.

## Introduction

According to the International Agency of Research on Cancer of the World Health Organization (WHO), primary liver cancer poses a serious threat to human health and survival. It was estimated that 906,000 new cases and 830,000 deaths occurred globally due to primary liver cancer in 2020 (1). Chronic hepatitis B virus infection is a major risk factor for the development of hepatocellular carcinoma (HCC), accounting for 43–80% of its total incidence etiologically (2, 3). In addition, prolonged HBV replication promotes immune-mediated liver inflammation to gradually progress to cirrhosis, ultimately leading to HCC (4).

Hepatitis B virus (HBV) is very adept at combining its own and host mechanisms to manage viral load and enhance persistence (5, 6). A key tool during the process is regulating the expression of non-coding RNAs by manipulating the epigenetic environment including microRNAs, lncRNAs, and circRNAs. Non-coding RNAs are aberrantly expressed from the beginning of HBV infection to liver fibrosis/cirrhosis, and finally to HBV-related HCC. They could participate in the proliferation, apoptosis, epithelial–mesenchymal transition (EMT), invasion, and migration of HCC cells *via* different mechanisms, such as making impacts on transcriptional regulation in nucleus, protein interactions, alternative splicing, and competing endogenous RNAs, thus affecting tumor progression (7–9).

Bibliometric analysis is a scientific and quantitative research method for publications including co-word analysis, social network analysis, and cluster analysis. Quantitatively statistical analyses are utilized to summarize the progress of research topics, figure out the contribution of authors, journals, and institutions, and explore international hotspots with emerging trends (10, 11). VOSviewer^1^ is a software displaying the visual map of the co-occurrence relationship between keywords and researchers (12). In recent years, the bibliometric study has been applied to the summary of research progress on clinical medicine and biomedicine (13, 14).

To our knowledge, however, no report has referred to the analysis of research progress and trends with respect to non-coding RNA and HBV-related HCC. The present study began with downloading publications information from the Web of Science Core Collection (WoSCC) from 2003 to 2022, consisting of annual distribution, country, institution, author, source journal, co-occurrence, co-citation of keywords, etc. Bibliometric methods were adopted to analyze the research status and development trends about non-coding RNA and HBV-related HCC. Our study aimed to explore the current hotspots in this field, provide novel ideas and perspectives for future investigations, and also assist researchers and experts in determining research subjects and directions on this topic.

## Materials and methods

### Data collection and literature retrieval strategy

**FIGURE 1:**
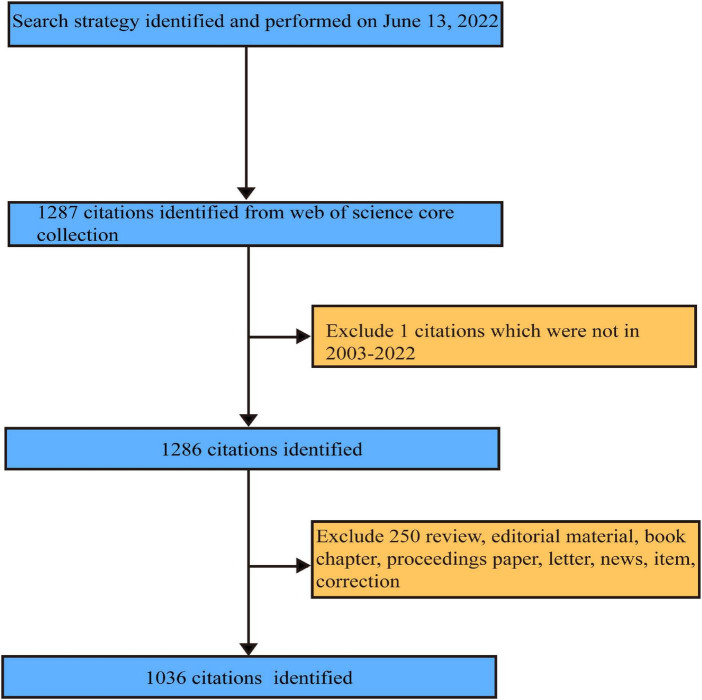
The flow diagram of literature selection according to the inclusion and exclusion criteria.

The literature on non-coding RNA and HBV-related HCC is available for download as “plain text” in WoSCC until 13 June 2022. Systematic literature retrieval was independently conducted by two investigators (Li-rong Yan and Ao-ran Liu). Ben-gang Wang made judgments on the divergence between two independent reviewers. Data collection and retrieval strategies are shown in Figure 1. Eligible publications met the following criteria: (1) search terms TS = [(“Hepatitis B Virus” OR “HBV” OR “hepatitis b viral” OR “hepatitis b” OR “hbv hepatitis” OR “chronic hepatitis b” OR “type b hepatitis”) AND (“Hepatocellular Carcinoma” OR “liver cancer” OR “HCC” OR “hepatic carcinoma” OR “hepatoma” OR “hepatocarcinoma” OR “hepatic cancer” OR “liver carcinoma”)] AND TS = (“Non-coding RNA” OR “microRNA” OR “microRNAs” OR “Circular RNA” OR “Circular RNAs” OR “long non-coding RNA” OR “long non-coding RNA” OR “lncRNA” OR “lncRNAs” OR “Small interfering RNA” OR “SiRNA” OR “Piwi-interacting RNA” OR “small nucleolar RNA” OR “ribosomal RNA” OR “transfer RNA” OR “small nuclear RNA” OR “tRNA-Derived Fragments” OR “tRNA halves” OR “miRNA” OR “circRNA” OR “tiRNA” OR “tRF” OR “miRNAs” OR “circRNAs” OR “miR*” OR “lnc*” OR “circ_*”); (2) article document types; (3) year of publication ranging from 2003 to 2022; and (4) information collected for each article containing publication, author, country, institution, journal, keywords, and citations.

### Data analysis and visualization maps

Bibliometrics is conducive to monitoring the development and patterns of available publications (11). Here, we extracted the literature information mentioned above from TXT files downloaded from WoSCC and then created a visual network map by means of VOSviewer Version 1.6.18. Datawrapper website was also employed for data visualization^2^. The most common bibliometric techniques are mainly composed of co-authorship analysis, co-occurrence analysis, and co-citation analysis. Co-authorship analysis may reveal the collaboration models among authors, institutions, and countries (15). Co-occurrence analysis could identify the relevance of multiple words by their frequency in the same article to manifest the hot topics and trends in a certain discipline. Co-citation analysis may help to establish the knowledge base of a discipline (16). These visualization maps make investigators identify bibliometric information such as active authors, institutions, countries, basic knowledge, research hotspots, and frontiers on a specific topic. The present study explored research hotspots about non-coding RNA and HBV-related HCC by co-word analysis for 100 keywords with the top frequency in retrieved publications. In the visual map of VOSviewer, each node was displayed by a circle with a label, and a larger circle represented higher frequency in co-occurrence analysis. The color of each circle was determined by the cluster it belonged to. The thickness and length of connection between nodes indicated their corresponding strength and relevance. A maximum of 1,000 connections in the network represented the 1,000 strongest relationships between nodes.

### Research ethics

All literature downloaded from WoSCC are part of public resource. The extraction of these data takes no account of humans or animals. Therefore, no ethical issue is involved in our data utilization, and the project has no need to be approved by an ethics committee.

## Results

### Annual global publication on non-coding RNA and hepatitis B virus-related hepatocellular carcinoma

**FIGURE 2:**
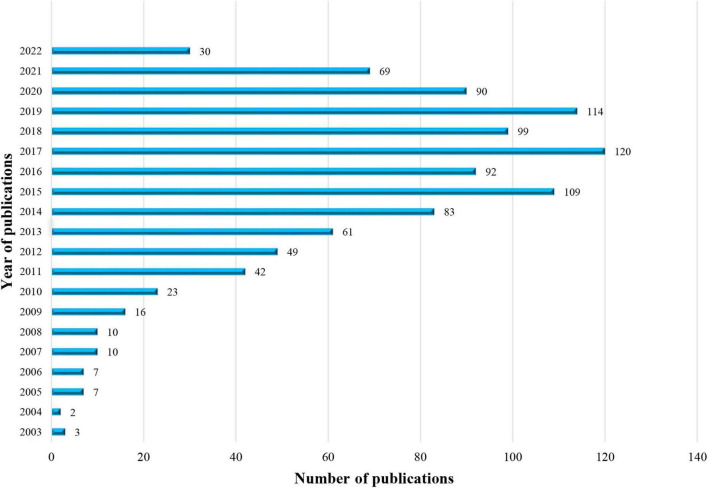
The number of articles about non-coding RNA and hepatitis B virus (HBV)-related hepatocellular carcinoma per year from 2003 to 2022.

A total of 1,036 articles about non-coding RNA and HBV-related HCC were retrieved from WoSCC. Annual global publications are shown in Figure 2. The publication on this topic has gradually increased during the past two decades. In 2003, only two related reports suggested that the subject had just begun to be focused. The published articles exceeded 50 for the first time in 2013 and exceeded 100 in 2015, maintaining around 100 in the following years. Hence, the research topic on non-coding RNA and HBV-related HCC remains novel with many problems to be solved and needs to be further investigated.

### Distribution of source journals and highly cited articles

Publications about non-coding RNA and HBV-related HCC were involved in 324 journals. The top 10 journals with the most articles are shown in Table 1, accounting for 24.4% (253/1036) of the whole. PLoS ONE had the most articles (*n* = 45) and 1,985 citations, followed by the World Journal of Gastroenterology (*n* = 33) and Scientific Reports (*n* = 32).

**TABLE 1:** The top 10 journals of publications on non-coding RNA and hepatitis B virus (HBV)-related hepatocellular carcinoma.

Rank	Source	Category	IF (2020)	Total publications (percentage)	Total citations
1	PLos One	Multidisciplinary sciences	3.240	45 (4.3%)	1,985
2	World Journal of Gastroenterology	Gastroenterology and hepatology	5.742	33 (3.2%)	1,073
3	Scientific reports	Multidisciplinary sciences	4.380	32 (3.1%)	837
4	Oncotarget	Oncology; cell biology	NA	30 (3.0%)	885
5	Hepatology	Gastroenterology and hepatology	17.425	29 (2.8%)	3,847
6	Oncology letters	Oncology	2.967	23 (2.2%)	339
7	Molecular medicine reports	Oncology; medicine, research, and experimental	2.952	20 (1.9%)	267
8	Biochemical and biophysical research communications	Biochemistry and molecular biology; biophysics	3.575	14 (1.4%)	637
9	Oncology reports	Oncology	3.906	14 (1.4%)	420
10	Cancer letters	Oncology	8.679	13 (1.3%)	526

Total citations of the 1,036 articles were 33,352 with a median of 22. The top 10 articles with the most citations are shown in Table 2, with a frequency ranging from 359 to 935. The article titled “Comprehensive analysis of microRNA expression patterns in hepatocellular carcinoma and non-tumorous carcinoma,” published on Oncogene in 2006 had the most citations of 935.

**TABLE 2:** The top 10 articles with the most citations.

Rank	Title	Journal	Citations	Avg citations	Pub. year	References
1	Comprehensive analysis of microRNA expression patterns in hepatocellular carcinoma and non-tumorous tissues	Oncogene	935	6	2006	(58)
2	Genetic landscape and biomarkers of hepatocellular carcinoma	Gastroenterology	654	16	2015	(59)
3	MicroRNA profiling in hepatocellular tumors is associated with clinical features and oncogene/tumor suppressor gene mutations	Hepatology	564	3	2008	(60)
4	Long non-coding RNA high expression in hepatocellular carcinoma facilitates tumor growth through enhancer of zeste homolog 2 in humans	Hepatology	552	5	2011	(61)
5	Inhibition of hepatitis B virus in mice by RNA interference	Nature Biotechnology	509	2	2003	(62)
6	Plasma microRNA panel to diagnose hepatitis B virus–related hepatocellular carcinoma	Gastrointestinal Cancer	436	4	2011	(63)
7	Circulating microRNAs, miR-21, miR-122, and miR-223, in patients with hepatocellular carcinoma or chronic hepatitis	Molecular Carcinogenesis	433	4	2011	(18)
8	TGF-β-miR-34a-CCL22 signaling-induced Treg cell recruitment promotes venous metastases of HBV-positive hepatocellular carcinoma	Cancer Cell	370	6	2012	(64)
9	MicroRNA-223 is commonly repressed in hepatocellular carcinoma and potentiates expression of stathmin	Gastrointestinal Cancer	363	2	2008	(65)
10	Serum microRNA profiles serve as novel biomarkers for HBV infection and diagnosis of HBV-positive hepatocarcinoma	Cancer Research	359	5	2010	(66)

### Distribution and co-authorship of country/region

**FIGURE 3:**
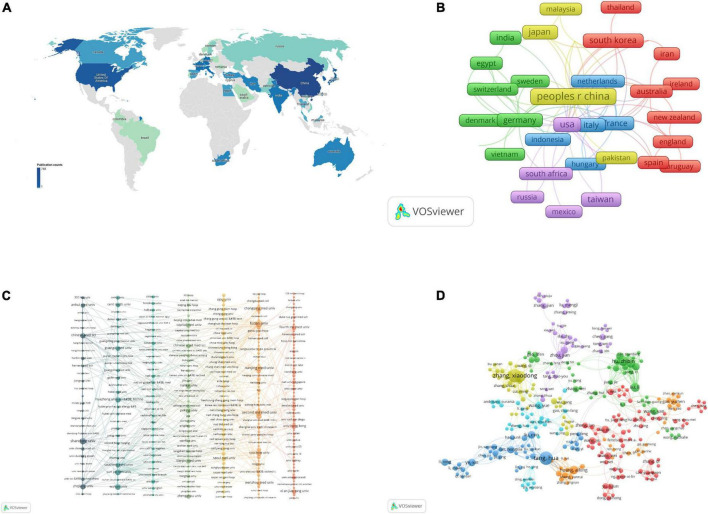
The co-authorship analysis of non-coding RNA and hepatitis B virus (HBV)-related hepatocellular carcinoma. **(A)** Distribution of country/region. **(B)** The co-authorship network of country/region. **(C)** The co-authorship network of the institution. **(D)** The co-authorship network of authors. Each node represents a country/region **(A,B)**, institution **(C)**, and author **(D)**, respectively. The size of nodes represents the publication number. The connection between nodes represents collaboration. The distance and thickness of the connection represent the relative strength of the relationship.

The subject of non-coding RNA and HBV-related HCC has been studied in 45 countries/regions worldwide (Figure 3A). The top 10 countries with the highest production are shown in Table 3. Among them, China had the most publications (748 publications and 23,184 citations) followed by the United States (133 publications and 8,380 citations) and India (49 publications and 3,164 citations). Co-authorship analysis of the country was performed by VOSviewer to demonstrate international collaboration in this field. The co-authorship network of countries is shown in Figure 3B, including 35 countries/regions of the whole. They were divided into five clusters represented by different colors. The largest cluster (red color) consisted of 10 countries centered on South Korea, Iran, and Australia. The United States had the most partners (*n* = 22) followed by Germany (*n* = 16), China (*n* = 14), Italy (*n* = 12), and France (*n* = 12).

**TABLE 3:** The top 10 productive countries/regions.

Rank	Country/Region	Publications	Citations
1	China	748	23,184
2	United States	133	8,380
3	Japan	49	3,164
4	South Korea	47	1,843
5	Taiwan	46	1,734
6	Italy	27	954
7	Germany	25	745
8	France	22	2,076
9	India	18	445
10	Singapore	17	899

### Distribution and co-authorship of institution

A total of 1,078 institutions were involved in the study for non-coding RNA and HBV-related HCC. The top 10 institutions with the most publications are shown in Table 4. Fudan University had the most publications (60 publications and 2,649 citations) followed by The Second Military Medical University (39 publications and 3,131 citations) and Nanjing Medical University (36 publications and 1,517 citations). We selected 327 institutions with at least two published articles. The co-authoring network was constructed for them by VOSviewer (Figure 3C), including 287 institutions. They were divided into seven clusters represented by different colors. The red cluster composed of 61 institutions was the largest one centered on the University of Hong Kong, The Fourth Military Medical University, and Inserm. The University of Hong Kong had the most partners (*n* = 17) followed by The Fourth Military Medical University (*n* = 14), Xi’an Jiaotong University (*n* = 12), and the National University of Singapore (*n* = 11).

**TABLE 4:** The top 10 productive institutions.

Rank	Institution	Publications	Citations
1	Fudan University	60	2,649
2	Second Military Medical University	39	3,131
3	Nanjing Medical University	36	1,517
4	Shandong University	36	1,188
5	Sun Yat-sen University	33	1,616
6	Wuhan University	33	954
7	Chongqing Medical University	29	1,066
8	Chinese Academy of Sciences	26	1,138
9	Huazhong University of Science and Technology	26	599
10	Nankai University	24	1,246

### Distribution and co-authorship of authors

A total of 6,154 authors participated in the retrieved 1,036 articles with an average of six authors in each article. The top 20 authors with the most publications are shown in Table 5. Zhang Xiao-dong (19 publications and 1,096 citations) and Tang Hua (19 publications and 547 citations) had the highest production followed by Ye Li-hong (15 publications and 977 citations). We selected 408 authors with at least three published articles and constructed the co-authorship network with 290 authors involved. They were grouped into seven clusters represented by different colors (Figure 3D). The 70 authors centered on Liu Jie, Li Wei, and Wang Wei formed the largest red cluster. Zhang Xiao-dong had the most partners (*n* = 25) followed by Tang Hua (*n* = 23) and Fan Hong-xia (*n* = 19).

**TABLE 5:** The top 20 productive authors.

Rank	Author	Total publications	Total citations	Per citations
1	Zhang, Xiaodong	19	1,096	58
2	Tang, Hua	19	547	29
3	Ye, Lihong	15	977	65
4	Hu, Zhibin	12	476	40
5	Liu, Li	11	311	28
6	Huang, Ailong	10	325	33
7	Wen, Juan	10	318	32
8	Zhou, Jian	10	939	94
9	Liu, Jibin	9	448	50
10	Liu, Jie	9	243	27
11	Liu, Min	9	284	32
12	Liu, Yao	9	315	35
13	Wang, Yan	9	348	39
14	Wang, Yue	9	741	82
15	Ye, Xin	9	444	49
16	Fan, Hongxia	8	407	51
17	Fan, Jia	8	748	94
18	Li, Ning	8	122	15
19	Wang, Wei	8	461	58
20	Wang, Yu	8	398	50

### Co-citation analysis of cited references

The retrieved 1,036 publications cited 25,975 articles in total. The top 10 articles with the highest citation frequency ranging from 50 to 137 are shown in Table 6. The article titled “MicroRNAs: genomics, biogenesis, mechanism, and function,” published on Cell in 2004 was cited the most. All the 25,975 cited articles originated from 2,555 cited sources including journals or books.

**TABLE 6:** The top 10 most co-cited references.

Rank	First author	Year	Journal	Title	Citations
1	David P. Bartel	2004	Cell	MicroRNAs: genomics, biogenesis, mechanism, and function	137
2	Hashem B. El-Serag	2007	Gastroenterology	Hepatocellular carcinoma: epidemiology and molecular carcinogenesis	93
3	Jian Zhou	2011	Journal of Clinical Oncology	Plasma MicroRNA Panel to Diagnose Hepatitis B Virus–Related Hepatocellular Carcinoma	70
4	Y. Murakami	2005	Oncogene	Comprehensive analysis of microRNA expression patterns in hepatocellular carcinoma and non-tumorous tissues	62
5	Saifeng Wang	2012	Hepatology	Loss of microRNA 122 expression in patients with hepatitis B enhances hepatitis B virus replication through cyclin G1-modulated P53 activity	58
6	Shunsuke Ura	2009	Hepatology	Differential microRNA expression between hepatitis B and hepatitis C leading disease progression to hepatocellular carcinoma	55
7	Anuradha Budhu	2008	Hepatology	Identification of metastasis-related microRNAs in hepatocellular carcinoma	53
8	Hashem B. El-Serag	2012	Gastroenterology	Epidemiology of viral hepatitis and hepatocellular carcinoma	53
9	Ladeiro Y.	2008	Hepatology	MicroRNA profiling in hepatocellular tumors is associated with clinical features and oncogene/tumor suppressor gene mutations.	51
10	Jin Hou	2011	Cancer Cell	Identification of miRNomes in human liver and hepatocellular carcinoma reveals miR-199a/b-3p as a therapeutic target for hepatocellular carcinoma	50

### Co-occurrence analysis of the top 100 keywords

The theme of publications is covered by their keywords; thus, high-frequency keywords are well suited for co-occurrence analysis. The top 100 keywords in this study were extracted and clustered by VOSviewer (Table 7). The co-occurrence network consisted of four clusters. Keywords were displayed in node labels and the size of each node represented their frequency. The connection between two nodes indicated the co-occurrence relationship between two keywords.

**TABLE 7:** Clusters of the top 100 Keywords.

Cluster	Keywords	Counts	Rank	Cluster	Key words	Counts	Rank
1	Cancer	243	5	2	Natural-history	17	87
1	Hepatitis B	194	7	2	p53	17	89
1	Biomarker	136	11	2	Cancer cells	15	93
1	Identification	100	15	2	Transgenic mice	15	97
1	Diagnosis	80	20	2	Viral-hepatitis	15	98
1	Prognosis	76	23	3	Hepatocellular carcinoma	731	1
1	Progression	66	26	3	Cell proliferation	222	6
1	microRNA-122	59	28	3	Metastasis	148	10
1	Survival	48	31	3	Apoptosis	113	13
1	Circulating microRNAs	40	37	3	Long non-coding RNA	102	14
1	Target	38	41	3	Growth	80	21
1	Serum	34	44	3	Invasion	80	22
1	Cirrhosis	32	46	3	Down-regulation	61	27
1	Inflammation	28	55	3	Migration	42	35
1	Alpha-fetoprotein	27	58	3	microRNA expression	33	45
1	Management	26	62	3	Overexpression	32	47
1	Therapy	25	65	3	Tumor-suppressor	32	48
1	Pathways	23	69	3	Promotes	31	51
1	Virus	20	75	3	Pathogenesis	28	56
1	Angiogenesis	19	76	3	Poor-prognosis	28	57
1	Plasma	19	78	3	Epithelial-mesenchymal transition	27	59
1	Microarray	18	82	3	Hepatitis	27	60
1	Profiles	18	84	3	Gastric-cancer	25	63
1	Prostate-cancer	17	90	3	Contributes	24	66
1	Prediction	16	92	3	Tumor-growth	21	71
1	Hulc	15	94	3	Colorectal-cancer	20	72
1	Recurrence	15	95	3	Tumorigenesis	20	74
1	Signature	15	96	3	C-myc	17	86
2	Hepatitis B virus	348	3	3	Non-coding RNAs	17	88
2	Hepatitis B virus X protein	160	8	3	Suppresses	17	91
2	Cells	114	12	3	miR-21	13	100
2	Replication	94	16	4	Expression	420	2
2	Activation	93	17	4	microRNA	284	4
2	Infection	91	18	4	Gene	153	9
2	Gene-expression	82	19	4	Risk	55	29
2	Liver	69	24	4	RNA	51	30
2	Protein	68	25	4	Breast-cancer	45	33
2	Up-regulation	47	32	4	Susceptibility	37	43
2	Hepatocarcinogenesis	45	34	4	Association	31	49
2	Transcription	42	36	4	Epidemiology	30	52
2	Mechanisms	39	38	4	Disease	29	53
2	Pathway	39	39	4	Methylation	29	54
2	Inhibition	38	40	4	Polymorphism	25	64
2	Nf-kappa-b	37	42	4	Biogenesis	23	67
2	DNA	31	50	4	Carcinoma	23	68
2	*In vitro*	27	61	4	Targets	23	70
2	*In vivo*	19	77	4	DNA methylation	20	73
2	Receptor	19	79	4	Lung-cancer	18	80
2	Mutations	18	83	4	Mechanism	18	81
2	siRNA	18	85	4	Promoter	14	99

**FIGURE 4:**
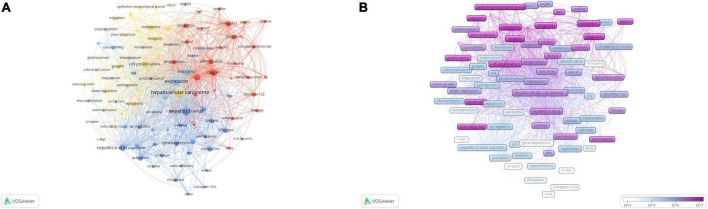
The co-occurrence analysis of non-coding RNA and hepatitis B virus (HBV)-related hepatocellular carcinoma. **(A)** The co-occurrence cluster analysis of the top 100 keywords. **(B)** The overlay map of the top 100 keywords. Each node represents a keyword. The size of nodes represents the publication number. The connection between nodes represents collaboration. The distance and thickness of the connection represent the relative strength of the relationship.

The keywords of hepatocellular carcinoma (731), expression (420), hepatitis B virus (348), and microRNA (284) took up the center of the visual network. All keywords were automatically grouped into four clusters based on similarity by VOSviewer (Figure 4A). The red cluster 1 represented non-coding RNA as a diagnostic and prognostic molecular marker for HBV-related HCC; the green cluster 2 represented abnormal regulation of non-coding RNA expression by hepatitis B virus X protein (HBx); the yellow cluster 3 represented the influence of non-coding RNA on malignant biological behaviors of HCC; and the blue cluster 4 represented the influence and epidemiological study of non-coding RNA on the risk of HBV-related HCC.

The evolvement trend of keywords was further explored and presented in a heatmap colored by VOSviewer based on their average appearing year (AAY) (Figure 4B). Cool colors represented keywords published earlier, while warm colors represented keywords published later. The most recent keywords included long non-coding RNA (AAY: 2018.1), epithelial–mesenchymal transition (AAY: 2017.9), migration (2017.8), invasion (AAY: 2017.6), prognosis (AAY: 2017.5), metastasis [(AAY: 2017.6) 2017.2], and biomarkers (AAY: 2017.2).

## Discussion

In the present study, we focused on the field of non-coding RNA and HBV-related HCC. Meanwhile, a bibliometric analysis was performed for the publications in WoSCC from 2003 to 2022, including the distribution of annual publication, country, institution, authors, source journals, co-occurrence of keywords, and co-citations. Furthermore, the development trends and current hotspots were also explored. Our study would provide novel ideas and perspectives for future investigations and also assist researchers and experts in determining the research subject and direction on this topic.

### Research trends in non-coding RNA and hepatitis B virus-related hepatocellular carcinoma

The research on non-coding RNA and HBV-related HCC has been gradually strengthened in recent years. The number of published articles showed a steady increase over the last two decades and was mainly concentrated after 2015, accounting for about 70% of the whole. They were involved in multiple disciplines. The top 10 journals sorted by publication volume consisted of five journals of oncology, two journals of gastroenterology and hepatology, two journals of multidisciplinary science, and one journal of molecular biology and biochemistry.

A total of 6,154 authors from 1,078 institutions in 45 countries/regions have published articles on non-coding RNAs and HBV-related HCC, suggesting that the topic has attracted widespread attention worldwide. Extensive cooperation has also been developed between countries/regions. China and the United States had the most published articles and formed the core of international cooperation. Therefore, China and the United States may currently grasp the frontiers of academic research in this field.

The top 10 institutions with the most published articles are all in China, suggesting that institutions in China are leading the way in terms of quantity. Moreover, 78% of research institutions in the collaborative network demonstrated extensive cooperation with each other.

Zhang Xiao-dong and Tang Hua were found to be the most productive authors in the study. Besides, Zhang Xiao-dong had the most collaborators. Only 290 of the 408 identified authors were included in the co-authorship network. We speculated that it might be due to some authors collaborating with authors outside the network.

### Research hotspots and emerging frontiers of non-coding RNA and hepatitis B virus-related hepatocellular carcinoma

The knowledge base and background of non-coding RNA and HBV-related HCC could be effectively revealed by co-citation analysis for cited references. The top 10 articles cited the most covered the studies in epigenetics, epidemiology, genetics, and molecular biology. Co-occurrence analysis of keywords could help to classify the main knowledge structure and hotspots. Here, co-occurrence cluster analysis was performed for the top 100 keywords, generating the four main clusters as follows.

#### Cluster 1: Non-coding RNA as a biomarker for hepatitis B virus-related hepatocellular carcinoma

Cluster 1 contained 28 high-frequency keywords such as hepatitis B, biomarker, diagnosis, prognosis, survival, and microRNA-122, suggesting that this cluster mainly focused on the biomarker study of HBV-related HCC. Early diagnosis of HCC is vital for the treatment outcome, and yet, effective markers remain lacking. It has attracted increasing attention for non-coding RNA to be a potential biomarker for the screening and early diagnosis of HBV-related HCC as well as the prediction of HCC prognosis.

A classification system based on seven miRNAs (miR-29a, miR-29c, miR-133a, miR-143, miR-145, miR-192, and miR-505) was suggested to have more significant sensitivity than alpha-fetoprotein (AFP) in distinguishing HCC cases from healthy controls, HBsAg inactive carriers, patients with chronic hepatitis B, and patients with HBV-cirrhosis. This miRNA-based classification was also the first biomarker established for the diagnosis of preclinical HCC and is expected to improve the clinical outcome of HCC through early diagnosis and precise treatment (17). MiR-122 is the most replicated miRNA biomarker in HCC with a sensitivity of 71–81%, a specificity of 59–83%, and an AUC of 0.63–0.87 in distinguishing HBV-related HCC from controls (18, 19). Recent studies showed that exosomal miRNAs might be better than whole-serum or plasma miRNAs for the early diagnosis of HBV-related HCC. In addition, the detection of miR-21 in exosomes was more sensitive than that in serum (20). Similarly, the exosomal miR-125b level of patients with HBV-related HCC was significantly lower than the serum level compared with patients with chronic hepatitis B or cirrhosis, which at least partially explained why the miR-125b level in exosomes, but not in serum, independently predicted HCC progression (21). In addition, exosomal miRNA levels of patients with HBV-related HCC had more significant differences than whole-serum compared with chronic hepatitis B or cirrhosis cases (22). The combination of miRNA with other classical serum markers can improve the sensitivity and specificity of early blood-based detection of HBV-related HCC (17, 23), especially for atypical HCC patients with low serum AFP levels. The expression levels of several miRNAs in liver tissue or circulation were shown to correlate with the severity and survival of patients with HBV-related HCC. For example, miR-223-3p expression in HBV-related HCC tissue was different from matched normal tissue. Circulating miR-223-3p might be a novel diagnostic and prognostic marker for patients with HBV-related HCC (24). Therefore, miRNAs are greatly potential markers for the diagnosis and prediction of HBV-related HCC.

In 2007, the expression of lncRNA HULC was first found to be significantly elevated in peripheral blood cells of 75% of patients with liver cancer (25). Subsequent research showed that the positive rate of HULC in the plasma of patients with HBV-positive HCC was higher than that of patients with HBV-negative, and the HULC level was correlated with the Edmondson score (26). These results suggested that plasma HULC was a potential biomarker for HBV-related HCC. The serum levels of lncRNA UC003WBD (27), AF085935 (27), UC00LNCR (28), and AX800134 (28) were also found to be elevated in patients with HBV-related HCC. In light of the above findings, lncRNAs may be useful biomarkers for predicting the risk and prognosis of HBV-related HCC.

Hsa_circ_0027089, a kind of plasma circRNA, was proven to distinguish HBV-related HCC from HBV-associated cirrhosis and healthy subjects, with AUC values of 0.765 and 0.794, respectively (29). It may be considered a biomarker for clinical diagnosis and evaluation of HBV-related HCC. In a microarray based on three pairs of HBV-associated HCC and adjacent tissue, aberrant expression of circRNAs was observed including 24 upregulated and 23 downregulated circRNAs (30). Hence, circRNAs may play key roles in distinguishing different types of HCC. For example, plasmatic circRNA can be used as a valuable diagnostic marker to distinguish HBV-related HCC from HBV-associated cirrhosis. Three circulating circRNAs (circ_0009582, circ_0037120, and circ_0140117) combined with AFP were reported to have higher sensitivity and specificity as potential diagnostic markers for predicting the development of HBV-related HCC (31). Zhu et al. found that plasma hsa_circ_0027089 could distinguish patients with HBV-related HCC from patients with non-HCC. Its combination with AFP had higher sensitivity to diagnose HBV-related HCC compared with patients with cirrhosis, healthy patients, and patients with no HCC but accompanied by poor specificity (29). Except for these, circRNAs can also be applied to clinical intervention targets.

In conclusion, non-coding RNA has great potential to become a marker for the diagnosis and prognosis of HBV-related HCC, which remains at the frontier of this field at present.

#### Cluster 2: Hepatitis B virus X protein dysregulates non-coding RNA in hepatitis B virus-related hepatocellular carcinoma

Cluster 2 contained 27 high-frequency keywords such as hepatitis B virus, hepatitis B virus X protein (HBX), replication, activation, hepatocarcinogenesis, and gene expression. Keywords in this cluster mainly reflected HBX, dysregulating the expression of non-coding RNA.

Hepatitis B virus X protein can affect miRNA biosynthesis, transcription, and translation in HBV-associated HCC (32), thus influencing tumor progression by altering the activity of tumor-related signaling pathways. Aberrantly expressed miRNA induced by HBx can change tumor signaling pathway activity affecting tumor progression. For instance, dysregulated HBx upregulates miR-21 (activated by IL-6) (33–35), miR-29, miR-155-5p (36), and miR-221/222 (37, 38) to inhibit the regulation of AKT/mTOR expression by PTEN. HBx also induces the upregulation of miR-21 to reduce the inhibition of WNT signaling by DCC6 (39) and enhances WNT signaling by decreasing PDCD4 expression and E-cadherin (33). Moreover, HBx downregulates let-7 to reduce its regulation on the activation of IL-6-induced JAK/STAT and mTOR-mediated transcription of oncogenic proteins such as C-MYC/MCL-1 (40–42).

In HBV-associated HCC, increased HBx level is associated with upregulated Zinc finger E-box binding homeobox 2 antisense RNA1 (ZEB2-AS1) level and the transition to mesenchymal phenotype. As an antisense lncRNA, ZEB2-AS1 forms RNA–RNA double strands by pairing with complementary bases at the 5′ splicing site of the precursor mRNA encoding ZEB2 (43). It prevents the binding of spliceosomes and keeps ZEB2 retained in mature transcripts by translating longer IRES-containing 5′ UTR, thus increased ZEB2 level inhibits E-cadherin transcription and promotes EMT. The upregulation of lncRNA UCA1 caused by HBX promotes cell growth and tumorigenesis by recruiting EZH2 and inhibiting the p27Kip1/CDK2 signaling pathway (44). HBX-associated lncRNA-ATB activated by TGF-β promotes the invasion and migration of HCC cells by inducing autophagy (45). The upregulation of MFG-AS1 caused by HBX promotes HCC cell proliferation and migration by enhancing MAFG expression and stabilizing non-myosin IIA (46).

In conclusion, dysregulated expression of non-coding RNA caused by HBX can significantly affect HCC progression. To date, research on this topic is mainly focused on miRNA and lncRNA, while circRNA and ribosomal RNA have not been involved yet, which provides us with some hotspots and blind spots, which is an important clue for selecting future research directions.

#### Cluster 3: Influence of non-coding RNA on the biological behaviors of hepatitis B virus-related hepatocellular carcinoma

Cluster 3 contained 26 high-frequency keywords such as hepatocellular carcinoma, cell proliferation, metastasis, apoptosis, growth, invasion, migration, epithelial–mesenchymal transition, hepatitis, and non-coding RNA. This cluster mainly reflected the influence of non-coding RNA on the biological behaviors of HBV-related HCC.

Non-coding RNAs can affect the biological behaviors of HCC *via* multiple mechanisms. First, lncRNAs participate in the transcriptional regulation in the nucleus. For example, urothelial cancer-associated 1 (UCA1) is a lincRNA with a length of 1,400 nt (47). HBx was found to upregulate UCA1 expression in HBV-related HCC samples and HBx protein-expressing cell lines (44), associated with increased cell proliferation and inhibition of apoptosis. Similar to many other lincRNAs, UCA1 can recruit EZH2 to mediate epigenetic silencing. The inhibition of its target, tumor suppressor p27, enables cyclin-dependent kinase 2 (CDK2) to accelerate G_1_/S cell cycle progression and cell proliferation. Some lncRNAs can also post-translationally regulate oncogenic or tumor suppressor proteins discovered in the nucleus and the cytoplasm. They enhance protein stability by directly binding to their target proteins or preventing ubiquitination and proteasomal degradation (48). The bound lncRNAs can also inhibit function or promote the degradation of proteins. The downregulated lncRNA-6195 in HBV-related HCC is a tumor suppressor that binds to the substrate-binding site (aa 237–405) of ENO1 to inhibit tumor growth (49). lncRNAs act as competing endogenous RNAs (ceRNAs) to sequester or sponge miRNAs to inhibit miRNA-mediated posttranscriptional gene silencing. The ceRNA network mediated by lncRNAs is closely associated with the initiation and development of HBV-related HCC. LncRNA PCNAP1 is a pseudogene of PCNA (50) highly expressed in HBV-positive HCC cells and tumor tissue (51, 52). PCNAP1 acts as a ceRNA to sponge the tumor suppressor miR-154, preventing it from repressing PCNA expression. Then, PCNA binds covalently closed circular DNA (cccDNA) by interacting with HBV core protein and promoting HBV replication and cccDNA accumulation. PCNAP1 was also reported to sponge the tumor suppressor miR-340-5p and prevent it from directly inhibiting ATF7 expression in HCC cells, promoting HCC cell proliferation (52). CircRNAs may play critical roles in the progression of HBV-related HCC through the circRNA/miRNA axis. For instance, the expression of circRNA_100338 was suggested to be associated with the metastasis of tumor cells. MiR-141-3p is a direct target of circRNA_100338 that regulates gene expression required for HCC development (53). Metastatic suppressor 1 (MTSS1) is a potential target of miR-141-3p that promotes HBV-related hepatoma cell migration associated with poor prognosis. Therefore, the circRNA_100338–miR141–MTSS1 network might exert oncogenic and pro-migratory roles in HBV-related HCC (54). In conclusion, the effects of non-coding RNA on the biological behaviors of liver cancer cells are mainly concentrated on proliferation, migration, invasion, and apoptosis, while other effects are less involved needing further exploration.

#### Cluster 4: Non-coding RNA and the risk of hepatitis B virus-related hepatocellular carcinoma

Cluster 4 contained 19 high-frequency keywords such as risk, susceptibility, epidemiology, polymorphism, biogenesis, and expression. This cluster mainly reflected the impacts of non-coding RNA on the risk of HBV-related HCC and associated epidemiological research. In a cohort of independent Han Chinese patients, the variant genotypes of rs7763881 in lncRNA HULC were proven to reduce the risk of HBV-related liver cancer (55). A genome-wide association study was conducted to investigate the loci associated with the risk of familial HBV-related HCC. It demonstrated that a group of SNPs overlapping with LINCO0272 was associated with an increased risk of HBV-related HCC, although the detailed molecular mechanisms were unclear (56). MiR-196a2 rs11614913 was also significantly associated with HCC susceptibility, especially in HBV-related HCC, and individuals carrying the TC/CC genotype were more susceptible (57). The findings showed that non-coding RNA variants could affect the susceptibility to HBV-related HCC.

### Emerging frontiers of non-coding RNA and hepatitis B virus-related hepatocellular carcinoma

The overlay map analysis for the top 100 keywords suggested that the research hotspots about non-coding RNA and HBV-related HCC in the recent 5 years were mainly focused on long non-coding RNA, EMT, migration, invasion, and other cellular biological behaviors. With the depth of research into the level of mechanism, lncRNA has been paid widespread attention as a hotspot molecule. However, circRNA and other biological effects of tumor cells remain less studied, which are valuable research directions.

### Limitations and expectations

In our study, a bibliometric analysis was performed to analyze the evolvement trends of research on non-coding RNA and HBV-related HCC comprehensively. Some limitations should be acknowledged. First, the literature enrolled in the study was only extracted from the WoSCC database, thus some relevant publications might be omitted. Second, no quality assessment was performed for included publications, all of which were considered to have the same validity. Third, some authors might use different spellings of their names or multiple names; thus, errors could be made during the automatic extraction of author names by VOSviewer.

Information from other databases such as PubMed and China National Knowledge Internet were less than WoSCC. It will be a concern to supplement our analysis with updated information from these databases timely, and data from other sources will also be addressed in future. Some new methods should be adopted to perform a weighted analysis for publications based on quality assessment.

## Conclusion

The study first comprehensively analyzed all publications on non-coding RNAs and HBV-related HCC with the aid of bibliometric analysis, including publication trends, global collaboration models, and research hotspots. The findings would help to identify new research subjects and frontiers as a guide to future directions.

## Data availability statement

The original contributions presented in this study are included in the article/supplementary material, further inquiries can be directed to the corresponding author.

## Author contributions

B-GW conceived and designed the study. L-RY and A-RL were responsible for the data analysis and performed data interpretation. L-RY, L-YJ, and B-GW wrote and revised the manuscript. All authors contributed to the article and approved the submitted version.

## Footnotes

1
http://www.vosviewer.com

2
https://app.datawrapper.de/

## Abbreviations

WoSCC: Web of Science Core Collection
HBV: hepatitis B virus
HBx: hepatitis B virus X protein
HCC: hepatocellular carcinoma
AAY: average appearing year
AFP: alpha-fetoprotein.
